# A pipelined, resource-efficient convolutional neural network architecture for detecting and diagnosing Alzheimer's disease using brain sMRI

**DOI:** 10.3389/fnins.2025.1653565

**Published:** 2025-10-15

**Authors:** T. Prasath, V. Sumathi

**Affiliations:** ^1^School of Electrical Engineering, Vellore Institute of Technology (VIT) Chennai, Tamil Nadu, India; ^2^Center for e-Automation Technologies and School of Electrical Engineering, Vellore Institute of Technology Chennai, Tamil Nadu, India

**Keywords:** Alzheimer's disease, Gabor transformation, data augmentation, CNN classifications, fuzzy C-mean

## Abstract

**Introduction:**

Alzheimer's disease (AD) is a progressive neurological disorder that impairs memory and cognitive function in elderly individuals. Early detection is vital to slow disease progression and enable timely therapeutic intervention. Traditional diagnostic approaches for AD, however, often involve high time complexity and significant computational resource utilization, highlighting the need for more efficient automated solutions.

**Methods:**

This study introduces a Resource Efficient Convolutional Neural Network (RECNN) framework for AD detection and diagnosis using brain MRI images. The methodology incorporates three main modules: Gabor transformation, data augmentation, and classification with an anomalous pixel segmentation algorithm. Gabor transforms are employed to enhance spatial frequency features and improve detection rates. Data augmentation techniques are applied to increase the diversity of training samples. The RECNN classifier is then used for image classification, and functional morphological segmentation is applied to classify affected pixels into mild or advanced stages of AD. Two benchmark datasets are utilized for training and testing the proposed framework.

**Results:**

The proposed RECNN-based system demonstrated superior detection and classification performance compared with conventional AD detection methods. The model achieved improved accuracy and robustness, with segmentation results enabling the differentiation between mild and advanced AD cases. Comparative evaluation confirmed that RECNN significantly reduces computational complexity while maintaining high diagnostic reliability.

**Discussion:**

The findings suggest that the RECNN framework offers a resource-efficient and accurate approach for AD detection using MRI data. By combining Gabor-based feature transformation, augmented data diversity, and advanced segmentation, the proposed method provides a scalable and clinically applicable tool for early diagnosis. Future work will extend the model to larger and more diverse datasets and explore hybrid architectures to further enhance diagnostic performance.

## 1 Introduction

Alzheimer's disease (AD) is a progressive neurodegenerative disorder that predominantly affects the elderly population. The disease often begins with subtle, asymptomatic changes, followed by a gradual decline in cognitive function. One of the earliest and most defining clinical features is impairment in memory function, which arises as healthy neurons progressively deteriorate. As the condition advances, many patients develop mild cognitive impairment (MCI), a transitional state between normal aging and dementia.

According to the Neuroimaging Society (NIS) 2021 report (Helaly et al., 2022; Jain et al., 2019; Wang Y. et al., 2018; Wang S. H. et al., 2018), AD is still incurable, even if detected at an early stage. However, timely diagnosis is considered crucial, as it allows for interventions that can delay disease progression and preserve quality of life. In particular, cognitive therapies and symptomatic treatments may help stabilize memory performance and slow the loss of functional independence.

Neuroimaging plays an essential role in AD detection and monitoring. Among the available modalities, Magnetic Resonance Imaging (MRI) and Positron Emission Tomography (PET) are the most widely adopted. While PET provides metabolic and molecular-level insights, its use is limited by the risk of radiation exposure. MRI, on the other hand, offers high-resolution structural information without the use of ionizing radiation, making it the preferred modality for longitudinal and clinical studies (Noor et al., 2020; Pulido et al., 2020; Altinkaya et al., 2020; Chaddad et al., 2018; Vatanabe et al., 2020).

MRI scans can be further divided into structural MRI (sMRI) and functional MRI (fMRI). Structural MRI provides superior pixel resolution and enables precise assessment of cortical and subcortical atrophy, which are key biomarkers of AD. Functional MRI, although lower in spatial resolution, captures dynamic neural activity and serves as a complementary tool for understanding disease-related brain dysfunction (Mahanty et al., 2024; Mandawkar and Diwan, 2024; Yao et al., 2023).

**Figure 1:**
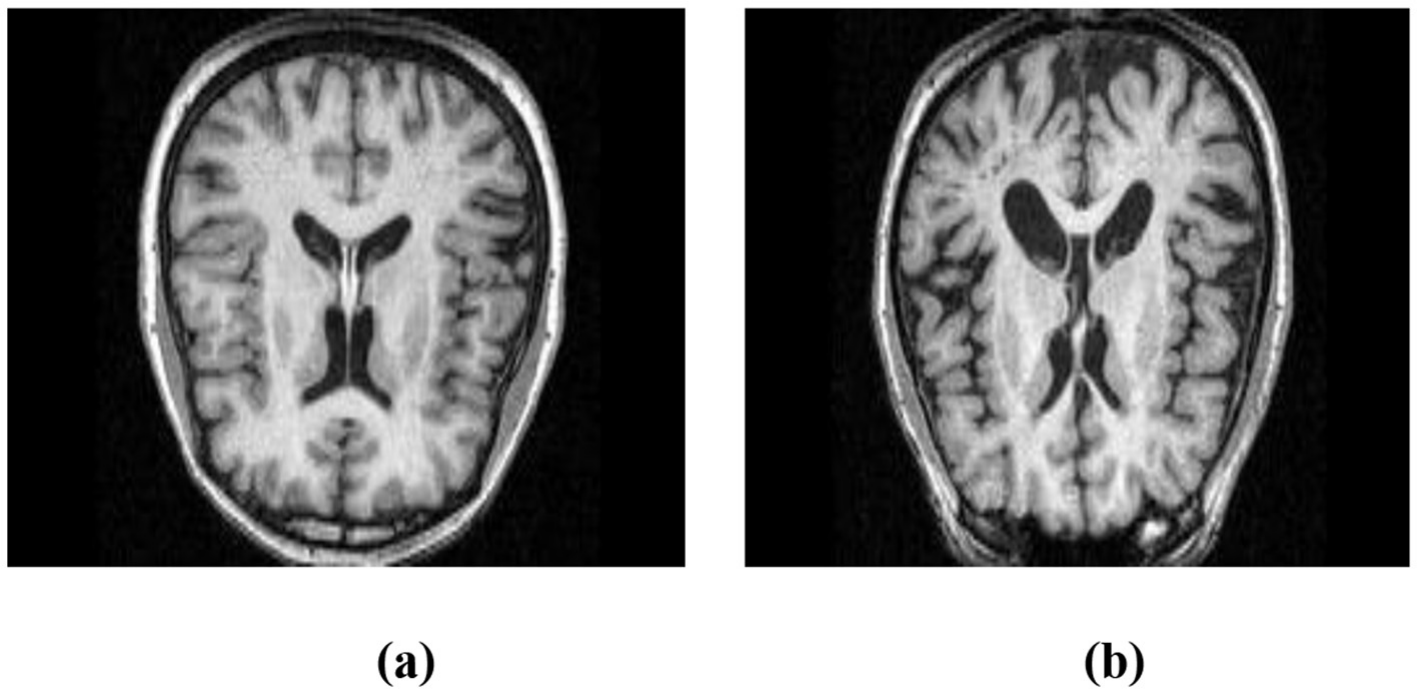
Structural MRI scans illustrating brain morphology. **(a)** Non-AD brain image. **(b)** AD brain image.

To demonstrate the utility of MRI in this context, Figure 1a shows a structural MRI scan of a healthy subject, while Figure 1b illustrates an AD-affected brain. Clear differences in structural integrity can be observed, highlighting the importance of MRI for early-stage detection.

Despite advances in imaging, traditional diagnostic approaches often fail to capture the diffuse and subtle structural variations that characterize early AD. Conventional CNN-based models used for image classification primarily rely on sequential feature extraction and may overlook weak but clinically significant signals. This limitation underscores the need for a resource-efficient and clinically adaptable deep learning framework.

In this study, we propose a Resource-Efficient Convolutional Neural Network (RECNN) that integrates Functional Gabor Transform (FGT) preprocessing, multi-path convolutional feature extraction, and Fuzzy C-Means clustering-based classification. By combining these innovations, the framework is designed to improve sensitivity to early-stage AD biomarkers while maintaining computational efficiency. Furthermore, the model not only distinguishes AD from non-AD cases but also provides a dual-stage categorization (mild vs. advanced), thereby enhancing its clinical applicability for assessing disease progression.

## 2 Literature survey

Recent years have witnessed a surge in interest in the application of deep learning and machine learning approaches for detecting and classifying Alzheimer's disease (AD) using neuroimaging data. Various strategies have been proposed, ranging from reinforcement learning frameworks to hybrid and hierarchical CNN-based models, each aiming to enhance accuracy and address the challenges of limited data, overfitting, and disease heterogeneity.

For instance, (Hatami et al. 2025) employed a deep reinforcement learning framework to improve AD image classification. Their non-linear learning strategy provided enhanced adaptability, yielding improvements in classification accuracy. Similarly, (Abhaya and Rajkumar 2025) proposed a hybrid architecture, Spike Google-Deep CNN SEViT, which successfully mitigated overfitting and produced reliable diagnostic outcomes. These contributions highlight the growing role of adaptive and hybrid methods in advancing AD detection.

Other studies have focused on traditional machine learning pipelines combined with neuroimaging. (Aberathne et al. 2023) utilized logistic regression on multimodal MRI and PET scans, reporting very high classification scores on benchmark datasets (KAD and MIRIAD). Along similar lines, (Diogo et al. 2022) introduced a multi-stage diagnostic framework based on support vector machines (SVM) with multi-kernel learning. By systematically varying kernel functions, their model demonstrated competitive sensitivity, specificity, and precision, highlighting the effectiveness of classical ML methods when carefully optimized.

Efficient architectures have also been explored. (Buvaneswari and Gayathri 2023) applied a linear variable model, achieving high accuracy with reduced computational demands. Meanwhile, (Liang and Gu 2020) incorporated weakly supervised deep learning with an attention mechanism to capture subtle disease cues, while (Lian et al. 2020) proposed a hierarchical CNN to localize atrophy patterns in MRI scans. Both approaches emphasized the importance of capturing pixel-level structural differences, with reported accuracies above 93% across multiple datasets.

Although these studies demonstrate remarkable progress, they share certain limitations. Many approaches prioritize overall classification accuracy but do not fully address the need for robust feature extraction across scales, reduction of overfitting in limited clinical datasets, or stage-specific diagnosis that could inform disease monitoring. Furthermore, while reported performance metrics are consistently high, these models are often tested on relatively homogeneous datasets, leaving questions about their generalizability to diverse clinical conditions, scanner types, and noise levels.

Building on these advancements, the present study introduces a Resource-Efficient Convolutional Neural Network (RECNN). Unlike prior research, RECNN integrates parallel multi-path convolution for rich feature extraction, introduces a feature integration stage to retain complementary signals, replaces dense layers with fuzzy C-means clustering to improve adaptability, and incorporates dual-stage diagnostic capability to separate mild and advanced AD cases. In doing so, it directly addresses the limitations identified in earlier studies and offers a unified, clinically meaningful framework for AD diagnosis.

In this study, we present a Resource-Efficient Convolutional Neural Network (RECNN) specifically designed for the classification of Alzheimer's disease (AD) images. The proposed framework introduces several distinct innovations that set it apart from conventional CNN-based approaches:

1. Multi-path convolutional design: the architecture incorporates parallel convolution–pooling streams that capture both fine-grained textural cues and broader structural patterns from brain images, enabling richer feature learning compared to traditional sequential CNN models.
2. Feature integration (FI) mechanism: features obtained from multiple abstraction levels are fused before classification, ensuring that complementary local and global information is preserved for more accurate detection of Alzheimer's-related changes.
3. Fuzzy C-Means (FCM)–based classification: instead of conventional fully connected layers, the model leverages FCM clustering to define adaptive decision boundaries, thereby improving robustness while mitigating overfitting in medical datasets with limited sample sizes.
4. Dual-stage diagnostic capability: beyond distinguishing AD from non-AD, the model provides additional diagnostic support by separating abnormal cases into mild and advanced stages, thereby enhancing its clinical utility for disease monitoring and progression assessment.

Together, these innovations establish RECNN as a technically distinctive and clinically meaningful framework for Alzheimer's disease classification.

The key contributions of this research are as follows:

- Functional Gabor transform (FGT): applied to capture spatial–frequency pixel properties, enhancing feature extraction from MRI images.
- Mitigation of overfitting: directional data augmentation (DA) strategies were employed during training to improve the model's robustness and generalization.
- Design of a novel RECNN architecture: a resource-efficient CNN framework specifically tailored for Alzheimer's disease (AD) image classification.
- Integration of Fuzzy C-Means (FCM): replaced traditional dense layers, providing adaptive decision boundaries and reducing overfitting risks.

## 3 Methodology

**Figure 2:**
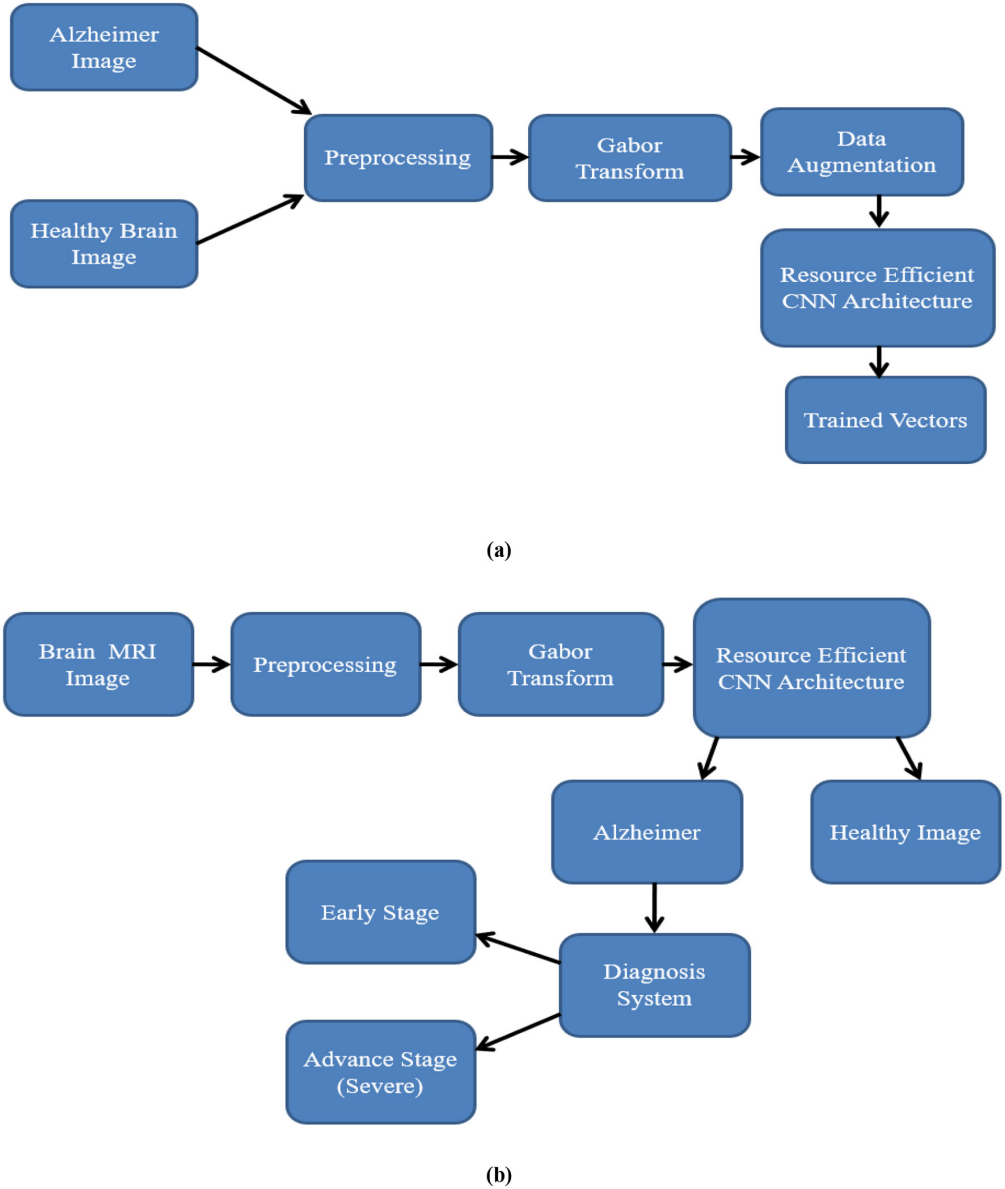
Workflow of the proposed RECNN during **(a)** training and **(b)** testing. In training, T1-weighted sMRI images are resized, Gabor-transformed, augmented, and then input into RECNN for learning. During testing, preprocessed images are fed into the trained RECNN for classification.

A RECNN-based deep learning approach is used in this method for the detection and diagnosis of AD images. Figures 2a, b illustrate the training and testing models. Brain MRI images of AD and non-AD subjects are obtained and preprocessed. The preprocessing module performs resizing and noise removal, followed by spatial frequency pixel property transformation using the functional Gabor transform. The data augmentation module in Figure 2a is used to increase the number of brain MRI images for training but is not applied during testing. As shown in Figure 2b, the testing stage classifies Gabor-transformed images with the RECNN architecture as either AD or non-AD. Subsequent diagnosis further categorizes AD images as mild or advanced.

The process involved in the training and testing stage is discussed as follows:

### 3.1 Preprocessing

In the preprocessing stage, images from the Kaggle^1^ and MIRIAD^2^ T1-weighted sMRI datasets were resized to a standardized resolution, ensuring uniformity across datasets and compatibility with the CNN input format.

### 3.2 Gabor transform

The brain MRI images obtained and utilized in this study exhibit spatial pixel representations in each image. The spatial variation of each pixel with respect to its surrounding pixels has a temporal relationship with the amplitude component, which reduces the detection rate of the AD image classification system. To address this, conversion from the spatial domain to the time and frequency domains with amplitude properties (Hosseini-Asl et al., 2016) is required to improve detection accuracy. To accomplish this, various conventional functional transformation models, such as Contourlet and Curvelet, are available; however, these models often result in a significant loss of pixel components during pixel property transformation. To improve the pixel property relationship during conversion, the functional Gabor transform (FGT) is used to perform spatial-frequency pixel property transformation.

The FGT has a unique two-dimensional kernel, which is multiplied by the two-dimensional brain MRI image to perform spatial-frequency pixel property conversion. The FGT kernel is given in Equation 1:


(1) $$\begin{array}{l} GK=exp\left(\left[\frac{-1}{2}\left\{{\left(\frac{x1}{s\;*\;x1}\right)}^{2}+{\left(\frac{y1}{s\;*\;y1}\right)}^{2}\right\}\right]\right)\\ \;*\;Cos\left(2\pi fx1\right) \end{array}$$

where *x*1 and *y*1 are the pixel coordinates in the brain MRI image, s is the spatial factor, and f is the frequency factor.

The spatial factor is set to 1, and the frequency factor is set to {1, 2, 3, and 4}.

The pixel coordinates in the brain MRI image are illustrated in the following Equations 2 and 3 with respect to its angle of orientation (θ).


(2) $$\begin{array}{l} x1=x\;*\;cos\theta +y\;*\;sin\theta \end{array}$$

(3) $$\begin{array}{l} y1=y\;*\;cos\theta -x\;*\;sin\theta \end{array}$$

where *x* and *y* are the pixel intensity values in the source brain MRI image with respect to the angle of orientation.

**Figure 3:**
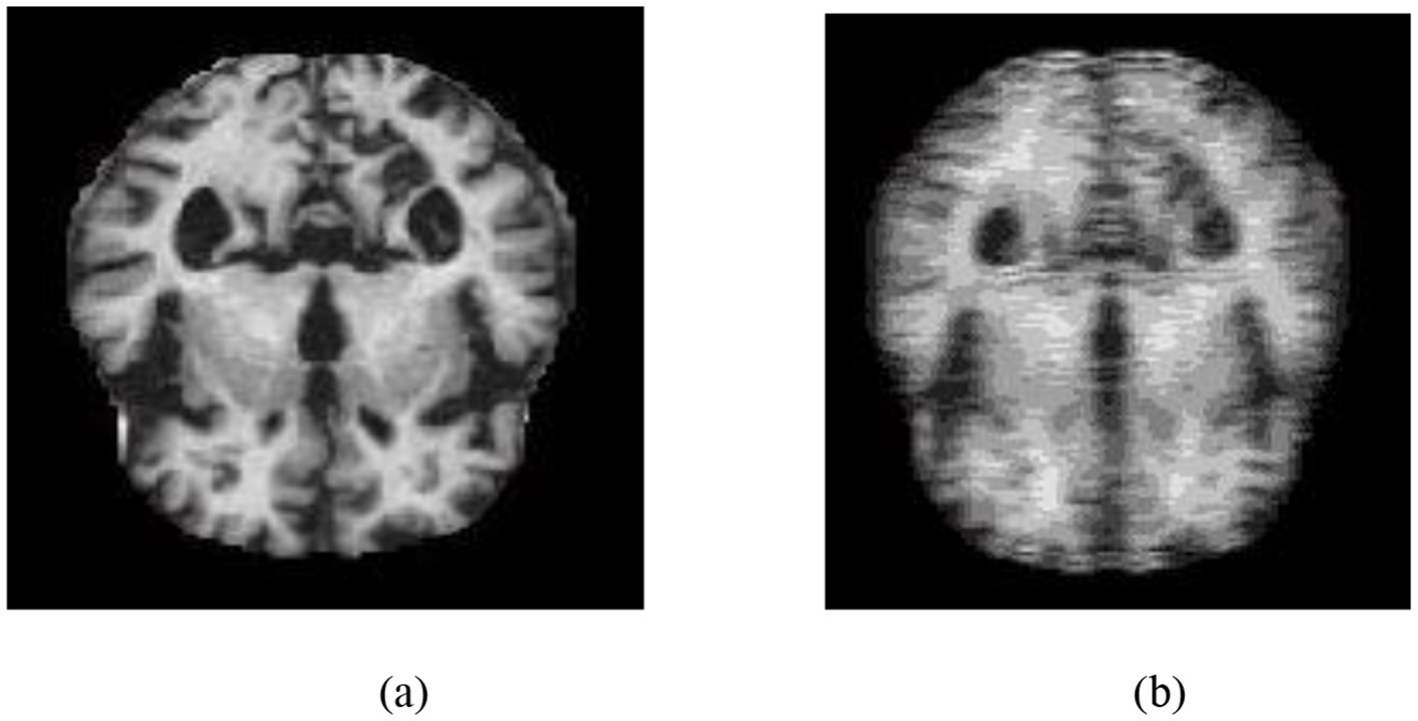
**(a)** T1-weighted sMRI image of an AD subject. **(b)** Corresponding Gabor-transformed image highlighting texture and frequency-domain features.

The angle of orientation of each pixel varies from −90 degrees to +90 degrees in 1-degree increments. Hence, for each frequency factor with a spatial factor, there is a 180-degree range of orientations, which creates 180 GK. These 180 GK are linearly convolved with the input source brain MRI image, producing 180 Gabor output images. These 180 Gabor output images are combined into a single Gabor image by selecting the maximum pixel intensity in each image. Figure 3 depicts an sMRI image of an AD subject and its corresponding Gabor-transformed image.

### 3.3 Data augmentation (DA)

**Figure 4:**
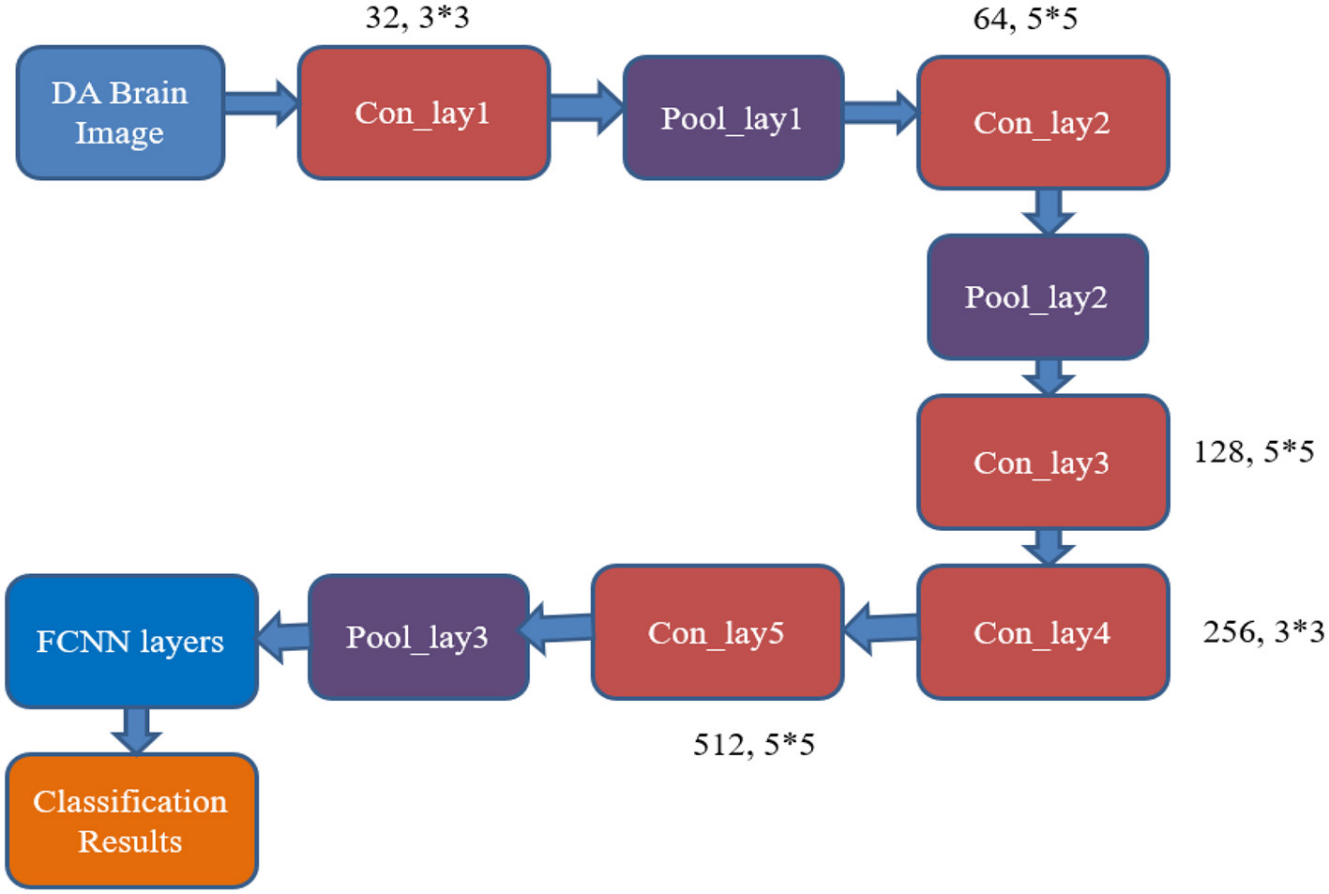
Data augmentation outputs illustrating directional transformations: **(a)** left-shifted image, **(b)** right-shifted image, **(c)** upward-shifted image, and **(d)** downward-shifted image. These augmented images are created to improve diversity during the training phase.

As part of the training pipeline, data augmentation was employed by applying directional translations to the input images, including shifts to the right, left, upward, and downward (Li et al., 2018), to increase dataset diversity and mitigate overfitting. Directional transformations were chosen because of their ability to maintain the anatomical integrity of brain areas while introducing spatial variation. Unlike other augmentation methods, such as random rotations, elastic deformations, or intensity-based modifications, directional translations preserve the alignment and structural consistency required for reliable AD classification using structural MRI. This method keeps the enhanced data clinically relevant while improving the model's robustness, generalization capability, and functional efficiency during training. The data-augmented images are shown in Figure 4.

### 3.4 RECNN architecture for AD classifications

This module presents the architecture of the proposed Resource-Efficient Convolutional Neural Network (RECNN), which is designed for classifying Alzheimer's disease using brain MRI images. The classification of a brain image as either AD or a healthy brain image is performed by the CNN classifier. The CNN classifier consists of a set of convolutional layers and pooling layers, along with neural network (NN) layers. The convolutional layers are designed with a set of filter kernels of varying sizes, and the NN layer has been equipped with various numbers of neurons to produce the desired output response.

**Figure 5:**
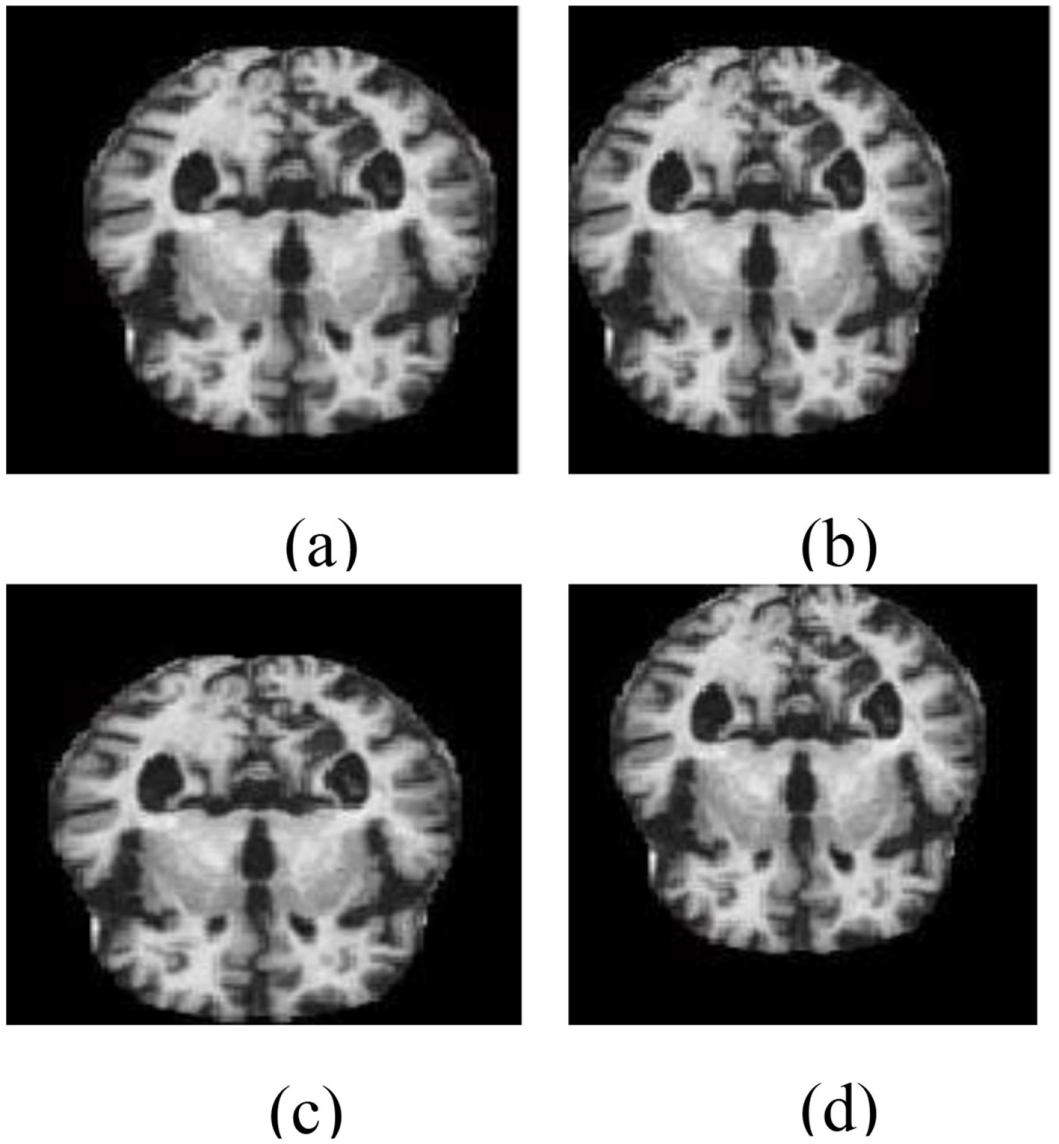
Architecture of the conventional AlexNet classifier used for AD image classification. The model processes brain MRI images through a series of convolutional, pooling, and fully connected layers for feature extraction and classification.

Figure 5 shows the existing CNN classifier for AD image classification (AlexNet). It includes five convolutional layers, three pooling layers, and a fully connected neural network (FCNN) layer.

The mathematical equations of the conventional classifier are as follows:


$$\begin{array}{l} Convolutional\;layers\text{ = }\{Con\_lay1\text{ : }Con\_lay2\;Con\_lay3\;\\ \text{ : }Con\_lay4\;Con\_lay5\}:\\ \;Con\_lay1=\text{32 filters and }{\text{3}}^{*}\text{3 filter kernel size}\;\\ \;Con\_lay2=\text{64 filters and }{\text{5}}^{*}\text{5 filter kernel size}\;\\ \;Con\_lay3=\text{128 filters and }{\text{5}}^{*}\text{5 filter kernel size}\;\\ \;Con\_lay4=\text{256 filters and }{\text{3}}^{*}\text{3 filter kernel size}\;\\ \;Con\_lay5=\text{512 filters and }{\text{5}}^{*}\text{5 filter kernel size}\;\\ \;Pooling\;layers=\{Pool\_lay1:Pool\_lay2:Pool\_lay3\text{\}} \end{array}$$

From Figure 5, it can be inferred that the desired output is obtained by utilizing a larger number of resources, like convolutional and pooling layers, with huge neuron requirements in the NN layer. This consumes more time and resources to produce the output responses. Hence, there is a need for developing a resource-optimized CNN architecture for the AD image classification process. The RECNN architecture is considered to overcome the limitations.

The proposed RECNN utilizes a pipelined framework to enhance the classification of AD from brain MRI images. This architecture combines both feature extraction and classification into a unified, continuous flow. Unlike conventional sequential systems—where extraction and classification are performed as independent processes—this method replaces the conventional Fully Connected Neural Network (FCNN) with a Fuzzy C-Means (FCM) clustering technique, ensuring a more cohesive and optimized flow of operations. Based on the streamlined architecture, the model is expected to reduce computational load and latency, thereby improving efficiency, execution speed, and scalability. This makes the RECNN particularly suitable for real-time analysis in clinical AD diagnosis scenarios.

**Figure 6:**
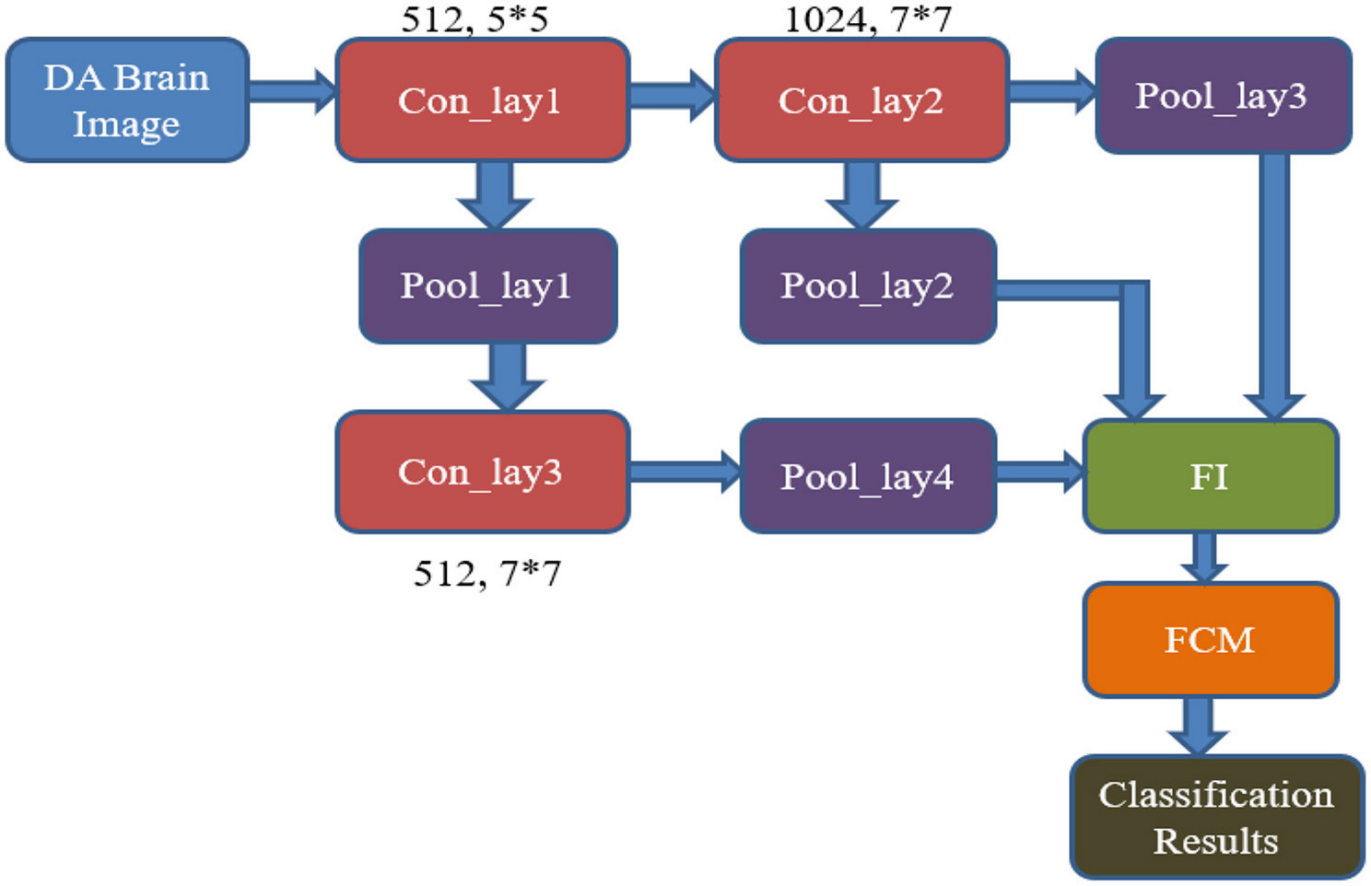
Architecture of the proposed RECNN for AD image classification.

The internal design of the RECNN classifier features three convolutional layers, four pooling layers, and concludes with an FCM-based classification module, as shown in Figure 6. The pipeline starts by feeding a data-augmented brain MRI image into the first convolutional block (Con_lay1).

- Con_lay1 applies 512 filters, each measuring 5 × 5, to perform basic feature extraction through convolution. The resulting feature maps are forwarded simultaneously to both Con_lay2 and the first pooling block (Pool_lay1).
- Pool_lay1 performs a max-pooling operation with a 2 × 2 kernel, down-sampling the feature maps to reduce spatial size and computational load.
- Con_lay2 consists of 1,024 filters and uses a 7 × 7 kernel. This layer is strategically placed deeper in the pipeline to extract complex and abstract spatial features, ensuring that early-stage layers remain computationally lightweight for all resource efficiencies.
- The output from Con_lay2 is processed by Pool_lay2 and Pool_lay3, both of which apply 2 × 2 max pooling to reduce dimensionality and retain prominent features.
- Simultaneously, the output from Pool_lay1 is passed into Con_lay3, which uses 512 filters and a 7 × 7 kernel to extract more refined patterns. These are then processed by Pool_lay4 using max pooling.

Outputs from Pool_lay2, Pool_lay3, and Pool_lay4 are then combined by a Feature Integrator (FI), which merges information across scales to create a rich representation of the brain scan.

The final stage involves the FCM classifier, which applies soft clustering techniques to differentiate between AD and non-AD (NAD) images. By replacing the traditional FCNN with FCM, the model gains improved interpretability and enhanced performance in handling uncertain or overlapping data distributions, which are common in early or borderline AD cases.

The steps involved in the FCM logical approach are as follows:

The integrated features are included in the new feature vector “*X*,”, which contains “*n*” features generated through the layers of the proposed RECNN architecture. The new feature vector is illustrated by the following equation.


(4) $$\begin{array}{l} x=\left\{x1,\;x2\ldots \ldots \ldots ..xn\right\} \end{array}$$

Each feature in “*x*” is assigned to a cluster, and the set of clusters is denoted in the following equation.


(5) $$\begin{array}{l} c=\left\{c1,c2\ldots ..ck\right\}, \end{array}$$

where *k* is the number of clusters that are assigned to the generated features.

The membership function “*M*” with *p* rows and *q* columns is represented by the following matrix.


$$\begin{array}{l} m=\left[\begin{array}{c} M_{1,1}\ldots \ldots ..M_{1,k}\\ M_{n,1}\ldots \ldots \ldots \ldots M_{n,k} \end{array}\right], \end{array}$$

where the following conditions need to be met to achieve the clustering functions.


$$\begin{array}{c} 0\leq M_{i,j}\leq 1\;\text{and}\\ \sum_{i,j}M_{i.,j}=1\\ 0\leq \underset{i,j}{\sum }M_{i.,j}\leq n\; \end{array}$$

**Step 1:** Assign *M* = [*M*_*i*., *j*_] with zero iterations.
**Step 2:** Center vector of the generated features:

(6) $$\begin{array}{l} {\text{C}}_{\text{ij}}=\;\frac{\sum_{\text{i},\text{j}}{\text{M}}_{\text{ij}}\;*\;{\text{x}}_{\text{i}}}{\sum_{\text{i},\text{j}}{\text{M}}_{\text{i}.,\text{j}}} \end{array}$$

**Step 3:** Update the membership function *M* using the following equation.

(7) $$\begin{array}{l} M^{ite}=\frac{1}{\left(\sum ||x_{i}-c_{i}{||}^{2}/||||x_{i}-c_{j}{||}^{2}||\right)} \end{array}$$

**Step 4:** The stop condition is fixed if the iteration of *M*^*ite*^ attains the maximum value.

### 3.5 Functional morphological algorithm

Functional Morphological Algorithms (FMA), when associated with pixel-level segmentation, enable the exact detection of localized brain alterations in MRI images. This enables reliable classification of Alzheimer's stages by revealing small structural differences in affected regions.

The stepwise procedure for Alzheimer's-affected pixel segmentation is outlined in Algorithm 1.

**Algorithm 1:** Functional Morphological Algorithm(FMA) for Alzheimer's-affected pixel segmentation.

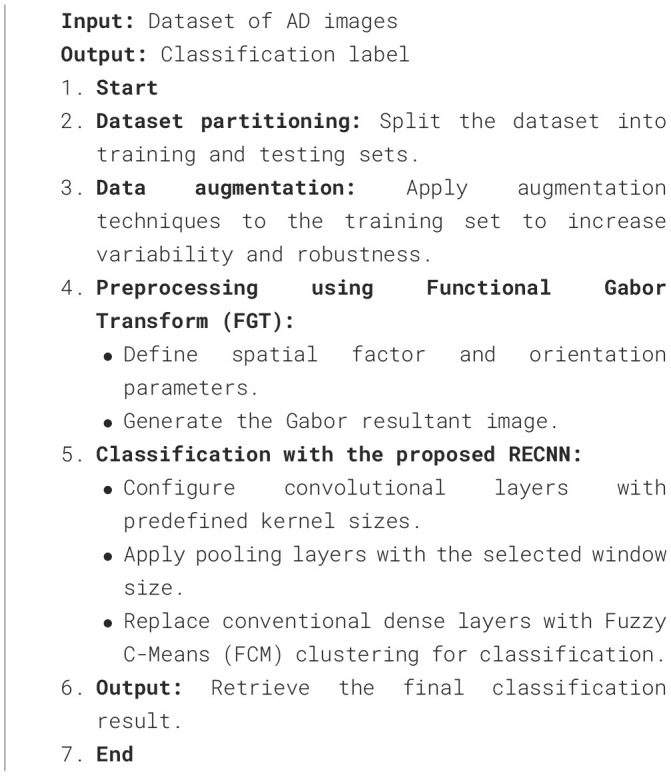

**Figure 7:**
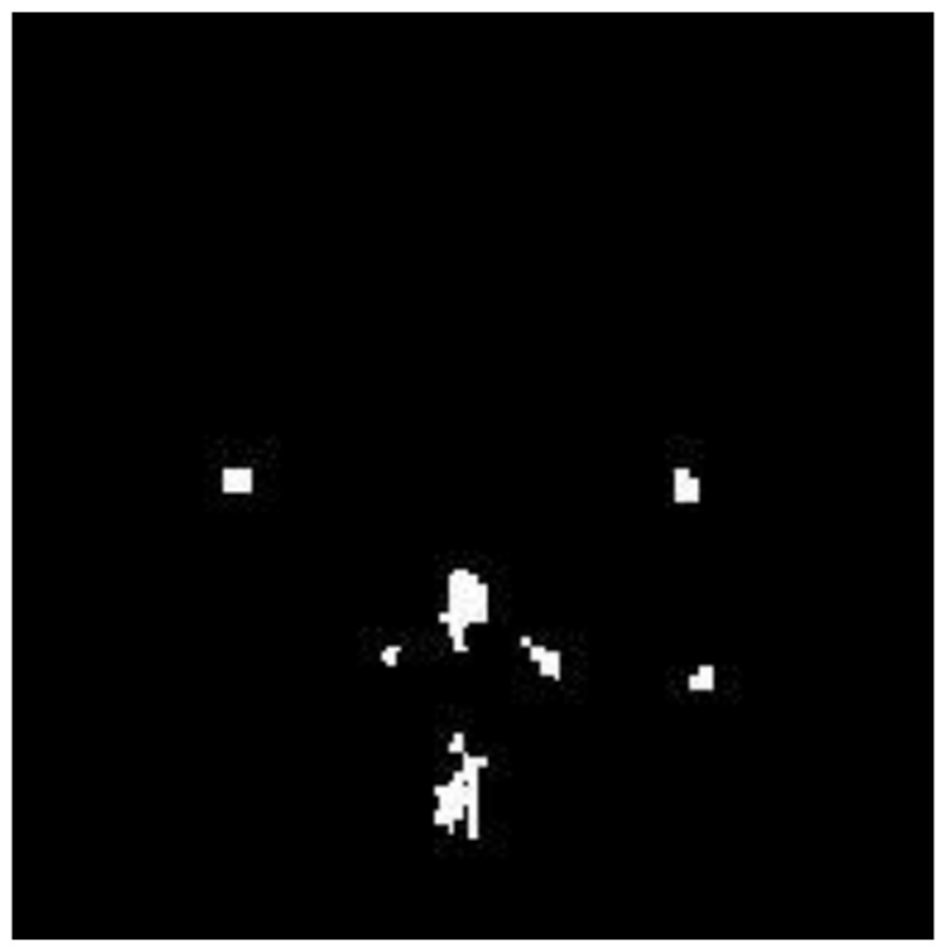
Segmentation output highlighting Alzheimer's-affected pixel (AP) regions in an AD brain image.

The FMA uses the detected AD image as its input image. Functional Morphological Dilation (FMD) is applied to the AD image using a disk-shaped structuring element with a radius of 0.5 mm, which increases or expands the outlier boundary of each pixel. The same image is then subjected to Functional Morphological Erosion (FME) using the same structuring element, which shrinks the outlier boundary of each pixel. AP pixels are detected and segmented in the AD image by subtracting the EI from DI. Figure 7 illustrates the segmentation of Alzheimer-affected pixel (AP) regions in an AD brain image, as classified by the RECNN model.

**Figure 8:**
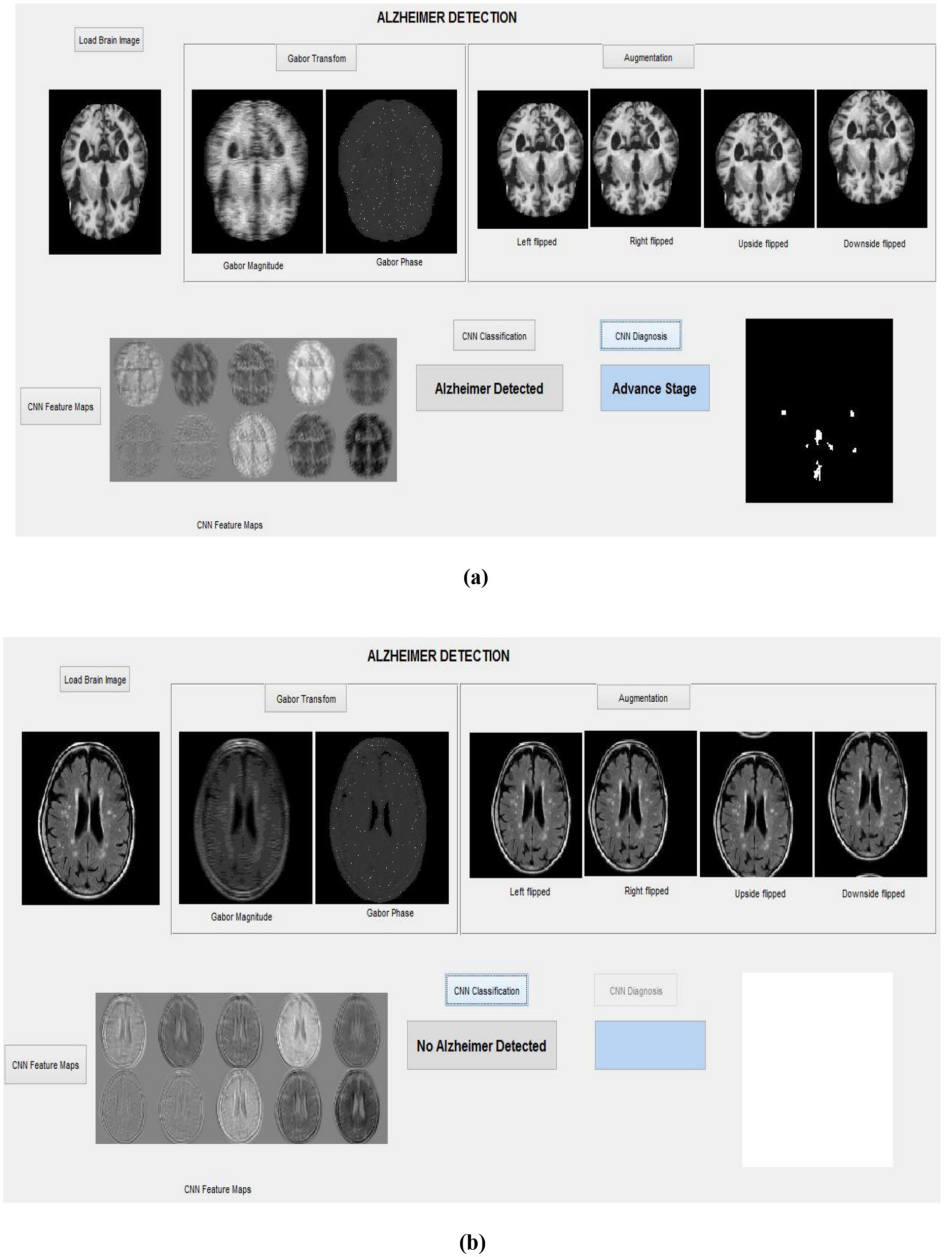
Simulation results with the graphical interface: **(a)** Detection of an advanced-stage AD image, **(b)** Classification of a non-AD (healthy) brain image.

These segmented AP regions in the classified AD image are input into the RECNN classification algorithm for further analysis. Specifically, AP pixels from mild AD images and AP pixels from advanced AD images are trained separately by the RECNN algorithm to produce individual training patterns (ITPs). During testing, the AP regions in the classified AD image are processed by the RECNN algorithm against the ITPs, producing an output classified as either mild or advanced AD. Figures 8a, b present the simulation results from the testing phase, illustrating detection and classification outcomes for both AD and non-AD images. The results from each module are visualized through a graphical user interface.

To illustrate the overall workflow of the proposed system, the complete process is summarized in Algorithm 2.

**Algorithm 2:** Proposed RECNN-based classification framework.

**Input:** Dataset of AD images
**Output:** Classification label
1. **Start** 2. **Dataset partitioning:** Split the dataset into training and testing sets. 3. **Data augmentation:** Apply augmentation techniques to the training set to increase variability and robustness. 4. **Preprocessing using Functional Gabor Transform (FGT):** • Define spatial factor and orientation parameters. • Generate the Gabor resultant image. 5. **Classification with the proposed RECNN:** • Configure convolutional layers with predefined kernel sizes. • Apply pooling layers with the selected window size. • Replace conventional dense layers with Fuzzy C-Means (FCM) clustering for classification. 6. **Output:** Retrieve the final classification result. 7. **End**

## 4 Results and discussions

The proposed Alzheimer's disease (AD) image detection and diagnosis framework based on the RECNN approach was evaluated using two publicly available datasets: Kaggle AD (KAD) and Minimal Interval Resonance Imaging in Alzheimer's Disease (MIRIAD). Both datasets are openly accessible and therefore do not require prior licensing agreements for research use. The KAD dataset includes MRI scans of both AD and non-AD subjects, each annotated by expert radiologists. It contains a total of 6,400 MRI brain images, of which 3,540 correspond to AD cases and 2,560 to non-AD cases. All images are provided at a uniform spatial resolution of 512 × 512 pixels.

In addition, this study also employed the MIRIAD dataset, curated by the Dementia Research Center (DRC) in the UK. This dataset comprises MRI scans from individuals aged 69–72 years, regardless of gender, with radiologist-provided annotations. The scans were obtained using a 1.5T Signa scanner with a 24 cm field of view and a 15° flip angle. The images are stored at a resolution of 256 × 256 pixels. In total, 8,500 images were used in this study, comprising 3,500 AD scans and 5,000 non-AD scans. All experiments and simulations were conducted in MATLAB R2024 on a Windows 10 Pro 64-bit system with an Intel Core i7-11700K processor (3.60 GHz, 8 cores/16 threads), 32 GB RAM, and 1 TB NVMe SSD. The inclusion of these two independent datasets was intentional, as they differ in both image resolution and acquisition settings. This diversity allows for a more rigorous evaluation of the robustness of the proposed approach.

The mathematical equations for calculating the performance of the AD image detection system are defined in Equations 8, 9:


(8) $$\begin{array}{l} AD\;Detection\\ Rate\;\left(ADDR\right)=\frac{Correctly\;detected\;AD\;images}{Total\;AD\;images} \end{array}$$

$$\begin{array}{l} Non-AD\;Detection\\ Rate\;\left(NADDR\right)=\frac{Correctly\;detected\;Non-AD\;images}{Total\;Non-AD\;images} \end{array}$$

Utilizing the equation mentioned above, the computational performance indicates a detection rate of 98.1% for ADDR and 99.1% for NADDR on the Kaggle dataset, while it reaches 99.7% for ADDR and 99.4% for NADDR on the MIRIAD dataset.

Furthermore, the performance of the AD detection and diagnosis system has been calculated and analyzed using the following mathematical equations:


$$\begin{array}{l} Sensitivity\;\left(Se\right)=\frac{TP}{TP+FN}\;\\ Specificity\;\left(Sp\right)=\frac{TN}{TN+FP}\;\\ Precision\;\left(pr\right)=\frac{TP}{TP+FP}\;\\ Accuracy\;\left(Acc\right)=\frac{TP+TN}{TP+TN+FP+FN}, \end{array}$$

where TP, TN, FP, and FN stand for true positive, true negative, false positive, and false negative, respectively. TP and TN indicate the correctly identified abnormal Alzheimer's pixels and healthy pixels. Incorrectly identified abnormal pixels and healthy pixels are represented by FP and FN, respectively.

Table 1 presents the experimental results of the computational performance of the AD image detection system on the KAD dataset. The proposed RECNN-based AD imaging detection and diagnosis system achieves 99.01%Se, 98.86%Sp, 98.89%pr, and 98.83%Acc, respectively.

**Table 1:** Computation of performance for the AD image detection system on the KAD dataset.

**Alzheimer's images**	**Se**	**Sp**	**pr**	**Acc**
A1	98.9	99.3	99.3	99.3
A2	98.0	99.1	99.1	99.1
A3	98.3	98.9	98.9	98.7
A4	99.2	98.5	98.4	98.5
A5	99.6	98.3	98.7	98.3
A6	99.4	99.2	99.3	99.7
A7	99.1	99.6	99.1	99.1
A8	98.9	98.1	98.9	98.6
A9	99.3	98.7	98.7	98.3
A10	99.4	98.9	98.5	98.7
Average	**99.01**	**98.86**	**98.89**	**98.83**

Table 2 presents the experimental results of the performance computation for the AD image detection system on the MIRIAD dataset. The proposed RECNN-based AD imaging detection and diagnosis system achieves 99.01%Se, 98.86%Sp, 98.89%pr, and 98.83%Acc, respectively.

**Table 2:** Computation of performance for the AD image detection system on the MIRIAD dataset.

**Alzheimer's images**	**Se**	**Sp**	**pr**	**Acc**
M1	99.3	98.9	99.3	98.4
M 2	99.1	99.3	99.1	98.2
M 3	98.9	99.1	98.9	98.9
M 4	98.4	99.5	98.5	99.3
M 5	99.2	98.6	98.6	99.1
M 6	99.7	98.9	99.3	98.8
M 7	99.1	99.3	99.1	98.5
M 8	98.6	99.1	98.9	98.3
M 9	98.9	98.7	98.4	99.2
M 10	99.3	98.4	99.3	99.1
Average	**99.05**	**98.98**	**98.94**	**98.78**

**Figure 9:**
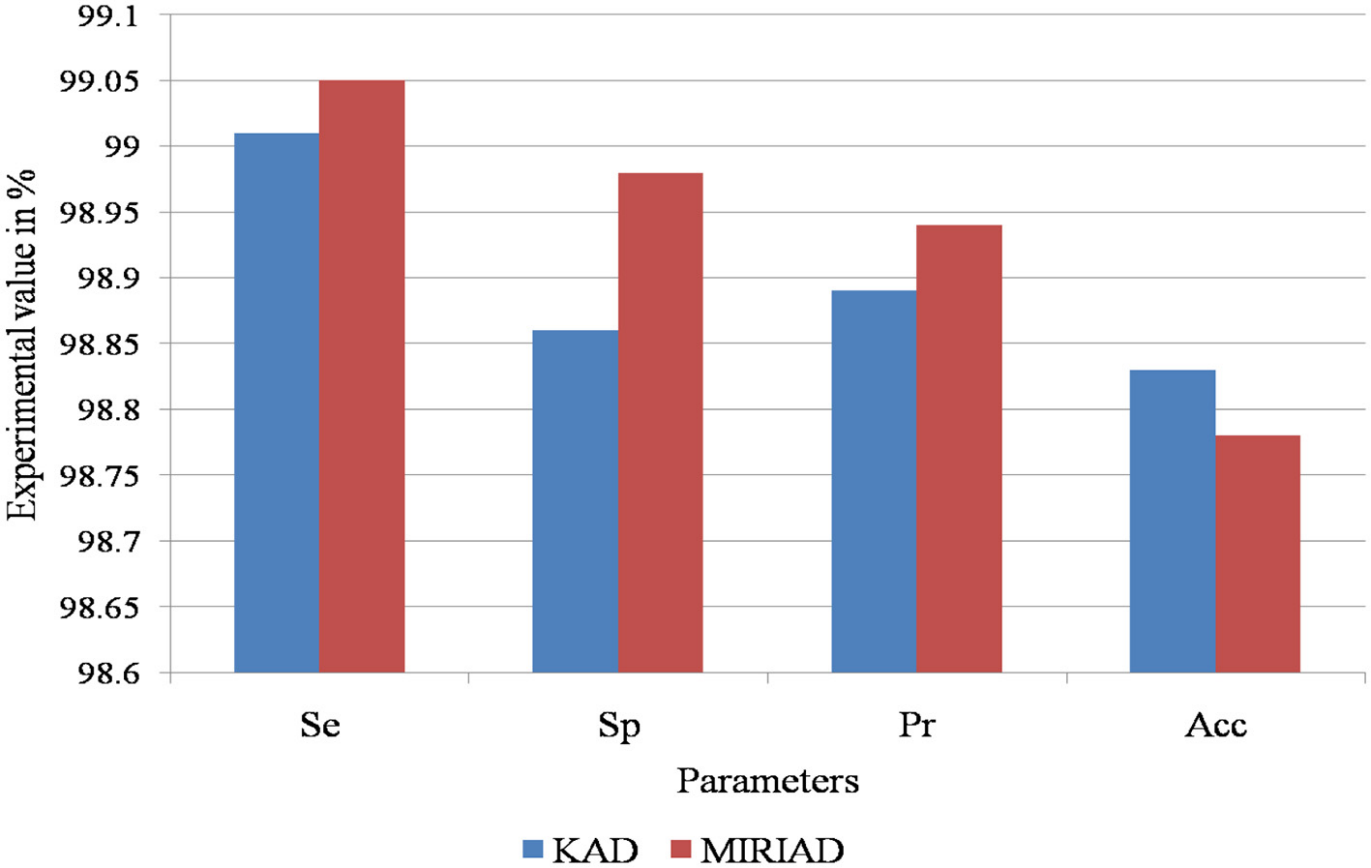
Performance evaluation of the proposed RECNN model on the KAD and MIRIAD datasets, illustrating classification metrics such as sensitivity, specificity, accuracy, and precision.

This study utilizes k-fold cross-validation as a robust statistical method to evaluate the model's performance across different assessment criteria. The computational analysis of the AD detection and diagnosis system using the RECNN approach on the KAD and MIRIAD datasets is shown in Figure 9.

Table 3 presents the computational analysis of the AD detection and diagnosis system using the RECNN approach.

**Table 3:** Computational analysis of the AD detection and diagnosis system using the RECNN approach.

**Performance computational parameters**	**KAD dataset**	**MIRIAD dataset**
**Se**	99.01	99.05
Sp	98.86	98.98
pr	98.89	98.94
Acc	98.83	98.78

Table 4 presents a comparative analysis of the proposed RECNN approach with other similar AD detection and diagnosis methods with respect to the AD images in the KAD dataset.

**Table 4:** Comparative analysis of the proposed RECNN approach with other similar AD detection and diagnosis methods with respect to the AD images in the KAD dataset.

**Methods**	**Performance computational parameters in %**			
	**Se**	**Sp**	**pr**	**Acc**
**RECNN approach**	**99.01**	**98.86**	**98.89**	**98.83**
(Aberathne et al. 2023)	97.32	97.56	97.57	97.43
(Diogo et al. 2022)	96.18	96.38	96.12	96.97
(Buvaneswari and Gayathri 2023)	95.97	95.10	94.56	94.57
(Liang and Gu 2020)	94.16	94.68	94.15	94.87
(Lian et al. 2020)	93.19	93.20	93.29	93.28

The experimental results of the proposed RECNN classification approach are compared with those of conventional AD detection methods in this study. (Aberathne et al. 2023) attained 97.32%Se, 97.56%Sp, 97.57%pr, and 97.43%Acc on the set of AD images on the KAD dataset. (Diogo et al. 2022) attained 96.18%Se, 96.38%Sp, 96.12%pr, and 96.97%Acc on the set of AD images on the KAD dataset. (Buvaneswari and Gayathri 2023) attained 95.97%Se, 96.10%Sp, 94.56%pr, and 94.57%Acc on the set of AD images on the KAD dataset. (Liang and Gu 2020) attained 94.16%Se, 94.68%Sp, 94.15%pr, and 94.87%Acc on the set of AD images on the KAD dataset. (Lian et al. 2020) attained 93.19%Se, 93.20%Sp, 93.29%pr, and 93.28%Acc on the set of AD images on the KAD dataset. The functional morphological algorithm proposed in this work, when integrated with the proposed RECNN classification algorithm, improves the overall performance efficiency with respect to the various evaluation parameters based on different datasets, as represented in Table 4. The data augmentation module in this proposed study enhances the dataset samples during the training stage of the classification algorithm, helping to eliminate overfitting. This is one of the key reasons for the improved experimental performance

Table 5 presents a comparative analysis of the proposed RECNN approach with other similar AD detection and diagnosis methods, specifically with respect to the AD images in the MIRIAD dataset.

**Table 5:** Comparative analysis of the proposed RECNN approach with other similar AD detection and diagnosis methods for AD images in the MIRIAD dataset.

**Methods**	**Performance computational parameters in %**			
	**Se**	**Sp**	**pr**	**Acc**
**RECNN approach**	**99.05**	**98.98**	**98.94**	**98.78**
(Aberathne et al. 2023)	97.46	97.93	97.57	96.38
(Diogo et al. 2022)	96.98	96.46	96.21	96.87
(Buvaneswari and Gayathri 2023)	96.35	96.10	96.47	95.56
(Liang and Gu 2020)	95.87	95.97	95.89	94.29
(Lian et al. 2020)	94.46	95.43	94.58	94.48

The experimental results of the proposed RECNN classification approach have been compared with the conventional AD detection methods in this paper. (Aberathne et al. 2023) attained 97.46%Se, 97.93%Sp, 97.57%pr, and 96.38%Acc on the set of AD images on the MIRIAD dataset. (Diogo et al. 2022) attained 96.98%Se, 96.46%Sp, 96.21%pr, and 96.87%Acc on the set of AD images on the MIRIAD dataset. (Buvaneswari and Gayathri 2023) attained 96.35%Se, 96.10%Sp, 96.47%pr, and 95.56%Acc on the set of AD images on the MIRIAD dataset. (Liang and Gu 2020) attained 95.87%Se, 95.97%Sp, 95.89%pr, and 94.29%Acc on the set of AD images on the MIRIAD dataset. (Lian et al. 2020) attained 94.46%Se, 95.43%Sp, 94.58%pr, and 94.48%Acc on the set of AD images on the MIRIAD dataset.

Based on the comparative analysis, it can be observed that the RECNN approach achieves better results than the other similar methods. Computational overhead refers to the consumption of computing resources for a specific task. The computational overhead includes computational time, memory utilization, bandwidth usage, and throughput. The determination of computational overhead is important for the Gabor transform and FCM due to their high computational cost and the large number of iterations. Table 6 presents the computational overhead of the proposed algorithm.

**Table 6:** Computational overhead of the proposed method.

**Computational overhead parameters**	**Modules**	
	**Gabor transform**	**FCM clustering**
Time consumption (ms)	0.71 for 180 orientations	0.42 for 100 iterations count
Memory (MB)	158.3	207.1
Throughput (b/s)	12,867	8,712

The execution time of the proposed method was evaluated in comparison with existing state-of-the-art approaches using the same computing environment. To ensure fairness, both the proposed framework and the baseline methods were implemented and tested on an identical simulation platform. The results demonstrate that the proposed approach achieves lower execution time, thereby reflecting better computational efficiency. A detailed comparison with recent state-of-the-art techniques is presented in Table 7.

**Table 7:** Execution time comparison with state-of-the-art methods.

**Authors**	**Execution time period (ms)**
**In this work**	**0.52**
(Aberathne et al. 2023)	1.67
(Diogo et al. 2022)	1.73
(Buvaneswari and Gayathri 2023)	1.87
(Liang and Gu 2020)	1.98
(Lian et al. 2020)	2.09

## 5 Conclusion

AD primarily affects healthy brain cells, leading to memory loss and dependence on others for daily activities. Slowing disease progression can help patients live more independently and improve their quality of life. The RECNN architecture proposed in this study detects and diagnoses AD images in an optimized manner, enabling early identification. Integrated with the Gabor transform and a data augmentation module, the RECNN architecture performs spatial transformations and enhances image samples, thereby improving classification rates. Using this framework, AD and non-AD images are classified with efficient resource utilization, while FMA further distinguishes mild and advanced stages of AD. The proposed RECNN-based AD detection and diagnosis system achieved 99.01%Se, 98.86%Sp, 98.89%pr, and 98.83%Acc on the KAD dataset, and 99.05%Se, 98.98%Sp, 98.94%pr, and 98.78%Acc on the MIRIAD dataset. The experimental outcomes of the RECNN method are significantly compared with other existing AD detection methods, confirming that the RECNN approach used in this study produces better results. This method may also be extended in future studies to identify and examine the impacts of AD on those with head injuries and brain tumors.

## Data Availability

The original contributions presented in the study are included in the article/supplementary material, further inquiries can be directed to the corresponding author.

## Footnotes

^1^https://www.kaggle.com/datasets/marcopinamonti/alzheimer-mri-4-classes-dataset (Accessed on Jul 2023).

^2^ https://www.ucl.ac.uk/drc/research-clinical-trials/minimal-interval-resonance-imaging-alzheimers-disease-miriad
